# Phylogeography and species limits in the red‐shouldered hawk (*Buteo lineatus*): Characterization of the Northern Florida Suture Zone in birds

**DOI:** 10.1002/ece3.5190

**Published:** 2019-04-24

**Authors:** George F. Barrowclough, Jeff G. Groth, William M. Mauck, Mary E. Blair

**Affiliations:** ^1^ Department of Ornithology American Museum of Natural History New York New York; ^2^ New York Genome Center New York New York; ^3^ Center for Biodiversity and Conservation American Museum of Natural History New York New York

**Keywords:** *Buteo lineatus*, hybrid zones, niche modeling, phylogeography, red‐shouldered hawk, suture zones

## Abstract

The North American red‐shouldered hawk,* Buteo lineatus,* is comprised of two widely allopatric eastern and western populations with an additional well‐marked subspecies in the Florida peninsula. The two eastern populations meet in northern Florida, the location of a well‐known suture zone in many nonavian organisms. We sequenced the complete mitochondrial ND2 gene and two nuclear introns to investigate its genetic population structure and species status. No mitochondrial haplotypes were shared between the eastern and western populations, and genetic variance among 14 populations was 0.42; almost all of this (0.40) was distributed among the three regions. A clade of haplotypes very common in the Florida peninsula decreased in frequency elsewhere and, when modeled as a hybrid zone, had an estimated width of 1,158 km with a center near Ocala, FL. Ecological niche modeling suggests the western, eastern, and Florida peninsula populations were geographically isolated during the last glacial maximum. We consider these to represent three phylogenetic species. A coalescent analysis incorporating incomplete lineage sorting and gene tree uncertainty also suggested the divergence between the western and eastern populations is consistent with species‐level divergence. With the addition of this hawk, four avian species are now known to hybridize along the Gulf Coast of the United States in or near the Northern Florida Suture Zone. The widths of these avian zones vary substantially (176–1,158 km) and appear to reflect magnitude of gene flow, rather than extent of genetic differentiation. None of these birds was suggested as possible exemplars in the original description of the suture zone. Of the six species that were so identified, three have been surveyed to date, but none of those was found to be genetically differentiated.

## 1 INTRODUCTION

Although generally congruent patterns are evident in the postglacial biogeography of Western Europe (Hewitt, 2000), this is less true of North America, particularly in birds (Zink, 1997). Patterns expected on the basis of palynological studies (Pielou, 1991) and other aspects of inferred Pleistocene history, such as the location of glacial refugia (Hubbard, 1973; Mengel, 1964; Remington, 1968) have not always been realized in subsequent phylogeographic investigations in this region (e.g., Bermingham, Rohwer, Freeman, & Wood, 1992). One general result in eastern North America, though, has been the repeated discovery of genetic differentiation across Gulf Coast river systems and the Appalachian Mountain chain in many disparate taxa, including plants, invertebrates, and both aquatic and terrestrial vertebrates (Avise, 2000; Soltis, Morris, McLachlan, Manos, & Soltis, 2006). These genetic divisions are proximal to Remington's (1968) Northern Florida Suture Zone, a proposed region of postglacial population convergence and interaction following dramatic landscape alteration during the Pleistocene (Emslie, 1998).

At the time of the Soltis' review, the sole avian exemplar of that Gulf Coast pattern was the Carolina chickadee (*Poecile carolinensis*: Gill, Slikas, & Agro, 1999). An additional species was known with marked genetic divergence in that region, the seaside sparrow (*Ammospiza maritima*). It possesses an east–west, not north–south, pattern, but this species is associated with salt marshes, rather than with forested habitat (Avise & Nelson, 1989).

As Barrowclough, Groth, Bramlett, Lai, and Mauck (2018) pointed out, the Gulf Coast pattern of genetic differentiation subsequently has been found in additional nonmigratory birds resident in eastern deciduous forests. However, the pattern was not observed in either of two grassland‐associated species, the northern bobwhite (*Colinus virginianus*: Williford, DeYoung, Honeycutt, Brennan, & Fernandez, 2016) and Bachman's sparrow (*Peucaea aestivalis*: Cerame, Cox, Brumfield, Tucker, & Taylor, 2014), nor in an open‐country species, the wild turkey (*Meleagris gallopavo*: Mock, Theimer, Rhodes, Greenberg, & Keim, 2002), or in a migratory species, the yellow‐throated warbler (*Setophaga dominica*: McKay, 2009), all of which were well‐sampled and are distributed throughout the Southeastern United States. Therefore, to further assess the importance of southeastern geographic features in avian differentiation during the Quaternary, it is apparent that nonmigratory, forest‐dwelling species are particularly appropriate for future investigation. One such species is the red‐shouldered hawk (*Buteo lineatus*).

**Figure 1:**
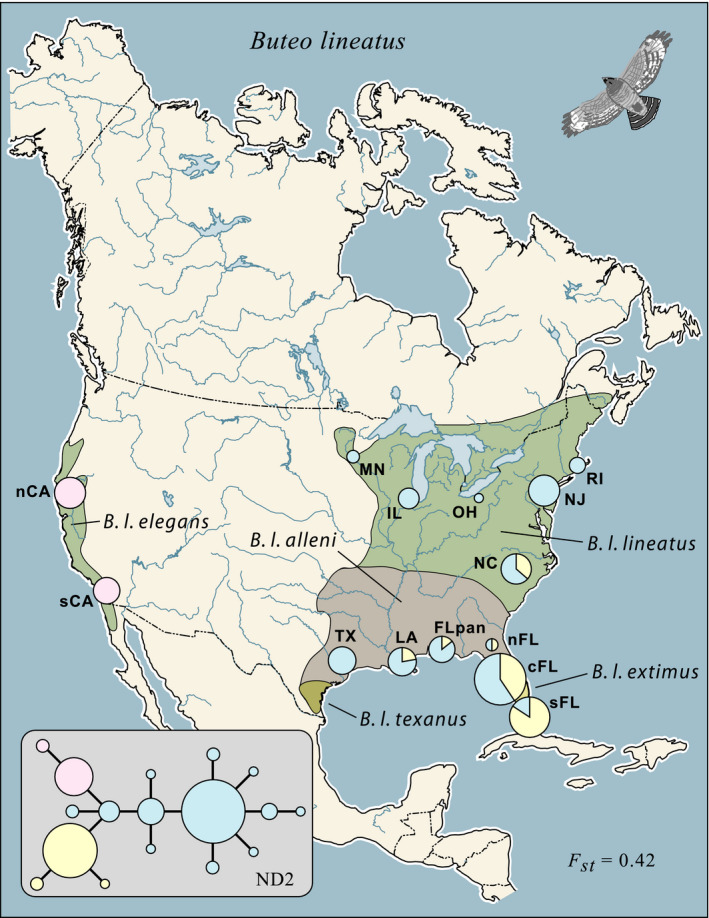
Network of observed ND2 haplotypes in *Buteo lineatus* and their geographic distribution; areas of haplotype pie diagrams are proportional to sample sizes. Approximate breeding distributions of five currently recognized subspecies are indicated

The red‐shouldered hawk is a generally common, sit‐and‐wait predator, especially associated with swamp and riparian habitats (Crocoll, 1994), with disjunct populations in eastern and western North America (Figure 1). In the east, it ranges from Maine and the Canadian Maritimes south to the Florida Keys and west to Minnesota and southern Texas; in the western United States. It is found west of the Sierra Nevada and Cascades in California and southwestern Oregon. It is a woodland hunter and a year‐round resident where climate allows. In eastern North America, only the most northern populations are migratory; the West Coast populations are year‐round residents.

Five subspecies (Figure 1) are presently recognized in two subspecies groups, four eastern taxa versus the populations on the West Coast (Dickinson & Remsen, 2013). The latter taxon, *B. l. elegans*, was originally described as a separate species (Cassin, 1855). Of the eastern subspecies, the Florida peninsula taxon, *B. l. extimus*, is the most distinctive. A prior phylogeographic survey using a small piece of mtDNA and 21 microsatellites found substantial divergence between the eastern and western population segments (Hull et al., 2008); however, most of the analysis was performed at the level of subspecies, rather than populations, potentially obscuring structure inconsistent with present taxonomy (Zink, 2004). In addition, the well‐marked Florida peninsula populations were not sampled.

We investigated phylogeography and species limits in the red‐shouldered hawk using population sequences of the entire mitochondrial ND2 gene and two nuclear introns taken from throughout its range. In addition, we used a variety of methods, including niche modeling and coalescent species delimitation, to investigate the evolutionary history of the hawk. Finally, we initiated quantitative comparisons of avian hybridization patterns in the Northern Florida Suture Zone as a step toward a general assessment of its importance in avian diversification during the Last Glacial Maximum.

## 2 METHODS

### 2.1 Samples

We attempted to obtain tissue samples of *Buteo lineatus* from throughout its known breeding range (Figure 1); all samples came from specimens cataloged in natural history museums, but many originally came from raptor rehabilitation centers (see Acknowledgments). We tried to obtain approximately ten unrelated individuals from within a relatively confined geographical region (up to several adjacent counties) to represent local populations. Along the Gulf Coast of the United States and in peninsula Florida, we used geographically denser sampling to investigate finer‐scale spatial variation.

### 2.2 Laboratory

We amplified and sequenced both mitochondrial and nuclear DNA sequences from the tissue samples. The laboratory work generally employed methods previously described (e.g., Barrowclough, Groth, Odom, & Lai, 2011; Barrowclough et al., 2018). We extracted total DNA from tissue samples using standard protocols; we used taq‐DNA polymerase and PCR methods to amplify the complete mitochondrial ND2 gene plus small fragments of the two flanking tRNAs using standard ND2 primers; the resulting products were isolated on agarose gels and reamplified as two overlapping fragments using internal primers. The fragments were sequenced in both directions using Sanger techniques, end‐labeled with fluorescent dye, and separated on an ABI 3730xl DNA analyzer.

Using similar techniques, we also sequenced two introns suggested to be useful for studies of avian phylogeography (Primmer, Borge, Lindell, & Saetre, 2002). These were intron 11 of the G3PDH gene (chromosome 1 in *Gallus*) and intron 5 of the TGFB2 gene (chromosome 3 in *Gallus*). Both of these loci were amplified and sequenced as single fragments using primers located in the two flanking exons. We designed novel primers for the G3PDH intron: G3P11Fb (5'‐TTCCAGGTTGTCTCTYGTGACTTCA‐3') and G3P12Ra: (5'‐CTTGGATGCCATGTGGACC‐3'). The primers used to amplify TGFB2 were previously described (Primmer et al., 2002).

The G3PDH and TGFB2 introns are autosomal and therefore diploid in birds; consequently, we encountered many individuals having ambiguous sites due to heterozygous alleles. We phased these alternate alleles in individuals having two or more ambiguities by sequencing the product of allele‐specific PCR amplification. Primers were designed to amplify one of the two alternate alleles in an individual by matching only one of these at the 3' end of the primer for one of the two (i.e., 5'‐most or 3'‐most) most distal ambiguities; in addition, the penultimate 3' base of the phasing primer was purposely mismatched to both alleles so that the sequence of the allele‐specific primer mismatched one allele at two 3' bases, including the final one, whereas the other allele was mismatched only at a single, penultimate base. After one allele was determined by sequencing, the alternate allele was reconstructed by subtracting the sequenced allele from the original, heterozygous sequence.

### 2.3 Sequence analysis

We assembled and edited sequences using SEQUENCHER software (Gene Codes). Mitochondrial sequences were trimmed of primer and flanking genes to provide complete ND2 sequences; the sequences were aligned by eye. We checked the sequences for quality by searching for indels, unexpected stop codons, heterogeneous base composition, and ambiguous sites.

For the purposes of estimating several population genetic parameters, individuals were assigned to one of 14 population samples (Figure 1). Standard genetic statistics were estimated for these populations using procedures previously described (e.g., Barrowclough et al., 2011; Barrowclough et al., 2018); these included Nei's (1987) nucleotide diversity and Tajima's (1989a, 1989b) *D* statistic. We used Excoffier, Laval, and Schneider's (2005) ARLEQUIN program to estimate various hierarchical genetic variance (*F*_st_) components (Wright, 1969). We used two ND2 sequences deposited in GenBank (*B. jamaicensis*: AY987156; *B. ridgwayi*: GQ264886) as outgroups and used PAUP* version 4.0b10 (Swofford, 2001) to infer gene trees and a minimum length network for the unique haplotypes.

The diploid G3PDH and TGFB2 sequences were also trimmed of primer and flanking exon sequence and aligned by eye. Individuals possessing two different alleles were phased, if necessary, as described above. The two alleles for each individual were assigned to the same 14 population samples used for the ND2 sequences and the same statistics computed. Possible recombination events were identified using a four‐gamete test, and sequences were trimmed to the largest sequence fragment consistent with no recombination. PAUP* was used to infer gene trees and minimum length networks for the unique alleles at each locus; in these analyses, we used the *B. jamaicensis* sequences as outgroups.

### 2.4 Hybrid zone analysis

We modeled the ND2 differentiation of *B. lineatus* populations in the southeastern portion of its range using a binary hybrid zone analysis. For a geographic axis, we designated a theoretical transect as a straight line from Key Largo, Florida north‐northwest to Lakeland, Georgia and then west along the 31° line of latitude through Texas. Each specimen of *B. lineatus* from Florida, Louisiana, and Texas was identified as possessing either a “Florida peninsula” or a “common” ND2 haplotype. We then projected each of the assigned haplotypes perpendicularly from its approximate original geographical position onto that transect. In this way, we obtained an ND2 haplotype frequency by distance survey through the southeastern portion of the hawk's range (e.g., Barrowclough et al., 2018).

Crank (1975) showed that transient neutral diffusion is expected to result in a modified cumulative normal distribution that scales with the square root of the product of the diffusion coefficient (gene flow) and time since primary contact (e.g., Barrowclough et al., 2018). In practice, hybrid zones and other clines are generally characterized by their maximum slope (May, Endler, & McMurtrie, 1975) or their 20%‐80% width (Endler, 1977). We fit the distribution of ND2 haplotypes along our designated transect to a cumulative normal distribution using the logistic regression procedure in SAS (1990). The form of the fit was *F*(*X*)* = *Φ(*mX + b*) where Φ is the cumulative normal distribution, *X* is the distance along the transect, and *m* and *b* are the maximum likelihood estimated parameters (slope and intercept). The center of the hybrid zone was estimated as *b/m*, the maximum slope as *m*/√(2π), and the 20%‐80% width as *1.68/m*; those estimates come directly from the definition of a normal distribution.

### 2.5 Coalescent species status analysis

We attempted to ascertain whether gene trees derived from our mitochondrial and nuclear loci might be consistent with more than a single species of red‐shouldered hawk under a coalescent process accounting for gene tree uncertainty, incomplete lineage sorting due to ancestral polymorphism, but no gene flow among populations (Yang & Rannala, 2010). We used the Bayesian Phylogenetics and Phylogeography Program (BPP version 3.3: Yang, 2015) to obtain joint Bayesian estimates of species delimitation and species trees by treating the ND2 and two nuclear genes as freely recombining among, but with no recombination within, loci. We assigned ND2 one‐quarter the effective population size of the nuclear loci. We treated sequences from California and those from the eastern United States as a priori candidate species, with *B. jamaicensis* sequences as a possible basal taxon.

We used the A01 MCMC algorithm (species delimitation with user‐defined guide tree) with adaptive adjustment during burn‐in of both priors and step length search parameters. Because the ages of the possible divergences (τ) and the ancestral effective population sizes (θ = 4μ*N_e_*) of these birds are poorly known, we used three sets of initial conditions for the runs. These were the following: large *N_e_* and old divergence (θ and τ gamma‐distributed priors of *G*(1,10) and *G*(1,10), respectively), small *N_e_* and recent divergence (priors of *G*(2, 2000) and *G*(2, 2000), respectively), and large *N_e_* and recent divergence (priors of *G*(1, 10) and *G*(2, 2000), respectively). Runs with each set of priors were repeated twice, with different initial seeds; the MCMC chains included 200,000 steps of burn‐in with adaptive parameter estimation and adjustment, followed by estimation chains 200,000 steps in length sampled every 20 steps (10,000 estimation points).

### 2.6 Ecological niche modeling

We attempted to estimate the approximate Last Glacial Maximum (LGM) distribution of *B. lineatus* using ecological niche modeling (ENM: Peterson et al., 2011); an approach that has been shown to be useful for interpreting phylogeographic patterns (Alvarado‐Serrano & Knowles, 2014). We created a file of latitudes and longitudes for *B. lineatus* using AMNH specimens, geo‐referenced museum specimens compiled in the Global Biodiversity Information Facility (GBIF.org) and documented breeding attempts in various state breeding bird atlases. We removed vagrants from the GBIF list by checking for specimens collected in United States and Mexican states and in Canadian provinces in which the hawk has not been documented to breed. Because *B. lineatus* is largely nonmigratory and its known migration does not extend south of its breeding range (Crocoll, 1994), this file represents a reasonable assessment of the species' breeding distribution.

We constructed ENMs using the WorldClim database for current climate (Hijmans, Cameron, Parra, Jones, & Jarvis, 2005) and the Paleoclimate Modelling Intercomparison Project Phase II database for the LGM (Braconnot et al., 2007). Based on the pattern of correlations among climatic variables, we reduced 19 commonly used bioclimatic factors to seven variables not highly correlated in the North American breeding range of *B. lineatus*. Two of these represented temperature extremes (minimum temperature of the coldest month of the year; mean temperature of the warmest quarter); three represented seasonality of precipitation (precipitation seasonality; precipitation of warmest quarter; precipitation of wettest quarter); one represented drought incidence (precipitation of driest quarter); and one representing frost, ice, and snow conditions (precipitation of coldest quarter). These variables had correlations ranging from 0.14 to 0.86 (median 0.49), and the correlation patterns were similar in the current climate and LGM databases. By using only (abiotic) climate variables in our analysis, our ENMs represent projections of areas with suitable climate for *B. lineatus* rather than its true range, which would also require biotic factors, such as competition with other predators. Nevertheless, our climatic ENMs do allow us to make broad comparisons about changes in suitable regions for *B. lineatus* between current conditions and those at the LGM.

We built ENMs using MAXENT version 3.3.3e (Phillips, Anderson, & Schapire, 2006); MAXENT estimates the unknown probability density for a species' actual range by minimizing the relative entropy between the species' observed range and environmental conditions across the study area (Elith et al., 2006). We selected the regularization parameter for the MAXENT model by testing a range of values from one to four and determining which model resulted in the highest area under the curve (AUC) of the receiver operating characteristic (ROC) plot, calculated with one‐fourth of occurrence data set aside as test data (Radosavljevic & Anderson, 2014). We chose a regularization parameter of two. Other model parameterizations followed generic recommendations by the model developers (Phillips et al., 2006; Phillips & Dudik, 2008).

We evaluated the predictive capabilities of the model using a fourfold partitioning (Peterson et al., 2011). For each partitioning, we calculated the AUC. We also calculated the omission rate by generating binary predictions using a maximum training sensitivity plus specificity threshold and used a binomial test to assess whether the observed omission rate was better than expected compared with a random prediction (Anderson, Gómez‐Laverde, & Peterson, 2002).

### 2.7 Comparative biogeography

We used several quantitative approaches to compare our *B. lineatus* data to three previously known instances of avian genetic divergence and hybridization along the Florida peninsula/Gulf Coast region of the United States; those were *Strix varia*, *Melanerpes carolinus*, and *Poecile carolinensis*. We sequenced the complete ND2 gene for two specimens each of *S. varia* and *P. carolinensis* to obtain estimates of genetic divergence along the Gulf Coast in these species. In both cases, we used individual specimens known to possess southeastern and western haplotypes based on prior studies of their phylogeography (e.g., Barrowclough et al., 2011; Gill, Slikas, & Sheldon, 2005). The ND2 sequences of *Melanerpes carolinus* had been reported previously (Barrowclough et al., 2018). We computed the percent sequence divergence between the southeastern and north or western haplotypes for the 1,041 base pairs of the ND2 gene for *B. lineatus* and the three additional species.

We modeled the hybrid zones in *S. varia*, *M. carolinus*, and *P. carolinensis* in a fashion similar to that described above for *B. lineatus*. For *S. varia*, we used the mitochondrial control region haplotype frequency data reported in Barrowclough et al. (2011) to assign individuals sampled in populations along the Gulf Coast and in peninsula Florida to either southeastern or western haplotype clades. The resulting binary assignments were then projected onto the same transect used in the *B. lineatus* analysis and fit to a cumulative normal distribution. For *M. carolinus*, we used complete ND2 sequences fit to that transect as described previously (Barrowclough et al., 2018). For *Poecile carolinensis*, we used the assignment of Gill et al. (1999) of individuals to eastern and western clades of mitochondrial restriction fragments; those assignments were projected onto the Georgia to Texas portion of the *B. lineatus* transect (there were no *P. carolinensis* samples from peninsula Florida in the Gill et al. study) and fit to a cumulative normal. Thus, we obtained estimates of the centers and widths of four avian hybrid zones.

We searched the literature for reports of natal dispersal distances in *B. lineatus*, *S. varia*, *M. carolinus*, and *P. carolinensis*; we used those data to estimate a gene flow distance, the root‐mean‐square dispersal distance, relevant to the population genetics of clines and hybrid zones (e.g., May et al., 1975). Studies that provided estimates of individual dispersal distances or other statistics sufficient to allow the estimation of gene flow were used. Gene flow was estimated as the root‐mean‐square dispersal distance if the distribution of individual dispersal distances was provided (e.g., Endler, 1977; Rockwell & Barrowclough, 1987). For cases in which mean dispersal distance plus the standard error of the mean were reported, we estimated gene flow as √N*SE; this estimate follows from the definition of a standard error (*SE*). We scattered the estimates of gene flow, and of ND2 divergence, against estimates of hybrid zone width in order to determine whether those parameters might be correlated.

## 3 RESULTS

### 3.1 Mitochondrial DNA variation

We obtained complete (1,041 bp) ND2 sequences from 130 specimens of *B. lineatus* from 14 population samples distributed from Minnesota to Rhode Island south to Texas and Florida and, in the west, from both northern and southern California. The sequences have been deposited in GenBank (MK575607‐MK575736); those accessions include state and county data as well as museum voucher provenance for each specimen. The sequences varied at 16 sites (15 transitions and 1 transversion); 2, 2, and 12 of the substitutions were at 1st, 2nd, and 3rd positions, respectively. All four of the 1st and 2nd position changes resulted in amino acid substitutions (nonsynonymous). No ambiguous sites were observed in the sequences, nor were there any indels or unexpected “stop” codons. The sequences comprised 17 haplotypes whose relationships are shown in Figure 1. The placement of the root of the network using sequences from *B. jamaicensis* and *B. ridgwayi* as outgroups was ambiguous due to the existence of multiple equal length phylogenetic trees. The root alternated between a southern Florida haplotype and the center of the entire network.

The extent of intrapopulational variation is described in Table 1. For population samples greater than five individuals, nucleotide diversity was highest in the Southeastern United States and decreased to the north and west; there was little variation in the California samples. None of the estimates of Tajima's *D* statistic was significantly different from zero.

**Table 1:** Estimates of within‐population genetic variation at three genetic loci in *Buteo lineatus*

Population	Gene	Sample size	Number haplotypes (or alleles) observed	Nucleotide diversity
Northern California	ND2	11	2	0.0003
	G3PDH‐I11	20	2	0.0170
	TGFB2‐I5	22	3	0.0022
Southern California	ND2	8	1	0.0000
	G3PDH‐11	14	2	0.0190
	TGFB2‐I5	4	3	0.0031
Minnesota	ND2	2	2	0.0019
	G3PDH‐I11	4	3	0.0240
	TGFB2‐I5	4	3	0.0040
Illinois	ND2	5	3	0.0013
	G3PDH‐I11	10	2	0.0137
	TGFB2‐I5	10	4	0.0029
Ohio	ND2	1	1	0.0000
	G3PDH‐I11	2	2	0.0385
	TGFB2‐I5	2	1	0.0000
Rhode Island	ND2	3	3	0.0026
	G3PDH‐I11	6	3	0.0282
	TGFB2‐I5	6	4	0.0032
New Jersey	ND2	11	5	0.0010
	G3PDH‐I11	22	3	0.0191
	TGFB2‐I5	22	6	0.0041
North Carolina	ND2	11	5	0.0020
	G3PDH‐I11	20	4	0.0214
	TGFB2‐I5	20	6	0.0037
Texas	ND2	9	3	0.0004
	G3PDH‐I11	14	2	0.0190
	TGFB2‐I5	12	6	0.0046
Louisiana	ND2	9	5	0.0016
	G3PDH‐I11	18	4	0.0185
	TGFB2‐I5	18	6	0.0048
Florida panhandle	ND2	7	4	0.0013
	G3PDH‐I11	14	3	0.0194
	TGFB2‐I5	14	7	0.0057
Northern Florida peninsula	ND2	2	2	0.0029
	G3PDH‐I11	4	2	0.0256
	TGFB2‐I5	4	4	0.0071
Central Florida peninsula	ND2	32	4	0.0016
	G3PDH‐I11	60	3	0.0202
	TGFB2‐I5	62	7	0.0046
Southern Florida peninsula	ND2	19	4	0.0009
	G3PDH‐I11	34	3	0.0202
	TGFB2‐I5	28	7	0.0042

Although we observed 17 mtDNA haplotypes, twelve of these were only present in one to three individuals each; only three of the haplotypes were common, that is, present in more than 10% of the individuals sampled. Those three showed strong, statistically significant, geographical structure (Figure 1), reflected in our estimate of *F*_st_ of 0.42 among the populations with sample sizes of five or greater (Table 2). One common haplotype was found only in California; a second was predominant in southern Florida and less frequent north and west of there; the most commonly observed haplotype was nearly ubiquitous in the eastern portion of the range.

**Table 2:** Estimates of among‐population components of genetic variation (*F*_st_) in *Buteo lineatus* at three genetic loci

Hierarchical division	ND2	G3PDH‐I11	TGFB2‐I5
Among all populations	0.42**	−0.01	0.05**
Among 3 regions	0.40*	−0.01	0.10*
Among populations within regions	0.14**	−0.00	−0.03
Between California and Eastern North America	0.65*	−0.01	0.22*
Between Florida peninsula and eastern North America	0.18	−0.01	0.01

For population samples with *N* ≥ 5.

* *p* < 0.05.

** *p* < 0.01.

### 3.2 Intron variation

**Figure 2:**
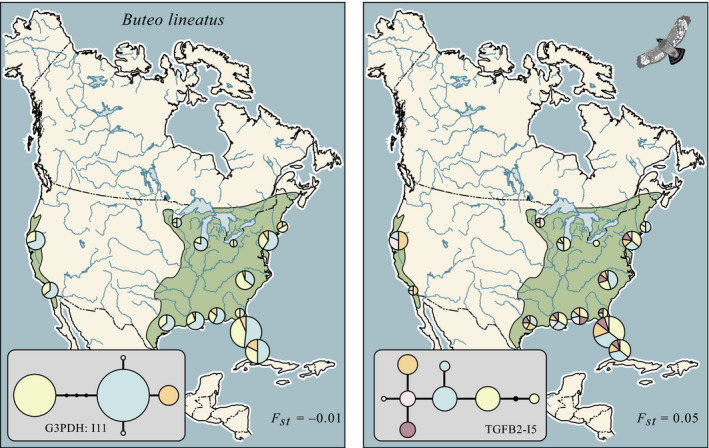
(a) Network and geographic distribution of observed G3PDH (intron‐11) alleles in *B. lineatus*. (b) Network and geographic distribution of observed TGFB2 (intron‐5) alleles. Areas of pie diagrams are proportional to sample sizes

The G3PDH‐I11 intron was 353 bp in length in *B. lineatus*. We obtained 242 sequences from 121 individuals from the same 14 populations sampled for ND2 (GenBank: MK523769‐MK524014). No indels were observed. After trimming the sequences to eliminate potential recombining fragments, we analyzed a sequence of 104 bp. Seven substitutions (three transitions and four transversions) resulted in five observed alleles; three of these were common, the other two occurred in only one or two individuals (Figure 2a). We were unable to place an unambiguous root on this network because two of the most divergent alleles observed in *B. lineatus* were also found in *B. jamaicensis,* probably as a result of incomplete lineage sortin*g*. Variation within populations at this locus is described in Table 1; one of the estimates of Tajima's *D* was significant, that for central Florida (*p* < 0.05). Geographic variation at this locus was very modest; one of the three common alleles was present at most eastern localities, but absent in California; the other two common alleles were found everywhere. Our estimate of *F*_st_ among populations with sample sizes greater than four was slightly negative and not statistically significant (Table 2).

The TGFB2‐I5 intron was 572 bp in length in *B. lineatus* (GenBank: MK551872‐MK552100). Again, no indels were found. We obtained 228 sequences from 114 specimens from the 14 populations. We analyzed 377 recombination free bases and found eight transitions (no transversions) that resulted in eight alleles; five of these occurred at frequencies greater than 10% (Figure 2b). The *B. jamaicensis* outgroup rooted the network at a common, widespread allele. All populations sampled were quite variable (Table 1) with the exception of Ohio (sample size of two). None of the estimates of Tajima's *D* was significant. Statistically significant interpopulation variation was found; the most common allele in the eastern portion of the range was not present in the California populations and neither was another common eastern allele. An allele present in both California samples at a frequency of 0.5 did not exceed a frequency of 0.25 in the east. Our overall estimate of *F*_st_ for this locus was 0.05 (Table 2); between California and the East Coast, it was 0.22.

The ND2 divergence exceeded that found in the two nuclear introns both between the California and eastern United States populations and among the eastern populations (Table 2). This is the expected pattern given the differences in effective population size between maternally inherited and autosomal loci (Irwin, 2018; Zink & Barrowclough, 2008).

### 3.3 Ecological niche and the last glacial maximum

**Figure 3:**
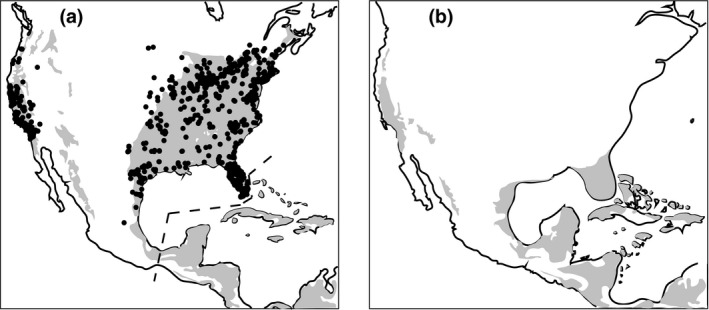
Ecological niche model for *B. lineatus*. (a) Solid dots represent breeding season training locations; dashed line indicates southern and eastern limit to training region; gray indicates MAXENT predicted current suitable habitat. (b) MAXENT predicted suitable areas (gray) during last glacial maximum

We found 2,764 museum specimen records or breeding bird atlas records for *B. lineatus* that possessed valid latitude/longitude data, given its known breeding season and range (Figure 3a). We trained our ENM and evaluated predictive abilities based on those data and projected the model onto a larger area that included the Yucatan peninsula and the Caribbean islands. Projection to this larger area facilitated comparison of climate suitability between the current climate and the LGM. The ENM projection for current climatic conditions (Figure 3a) thus shows *B. lineatus* suitability in areas where the species is not known to be present, perhaps because of competitive exclusion by some other large sit‐and‐wait predator (e.g., *Rupornis magnirostris*). The ENM projected to the LGM (Figure 3b) shows a considerable constriction in potential suitable habitat in the eastern United States compared with the current ENM projection. There was less change between predicted present and past suitable habitat in western North America, and there was no indication of potential LGM contact between the eastern and western portions.

Our ENM showed strong ability to predict the current observed localities, with AUC values >0.92 and significant binomial tests (range of AUC values 0.29–0.938, omission error 0.03–0.1 at *p* < 0.001). Variables with the largest percentage contribution to the ENM were mean temperature of the warmest quarter (43%) and precipitation in the coldest quarter (38%).

The projection of the ENM for *B. lineatus* suggests there was a gap in suitable habitat along the Gulf Coast during the last glacial maximum (Figure 3b), provided the niche of the hawk was conserved over evolutionary time (e.g., Peterson, Soberón, & Sánchez‐Cordero, 1999). If ancestors of *B. lineatus* occupied the Florida peninsula and Texas Gulf Coast portions of the suitable habitat at that time, then those allopatric populations would have been free to differentiate in size, plumage, and various DNA markers during the period of isolation. Subsequent contact and hybridization of the formerly allopatric population segments would have occurred as climate ameliorated and the intervening habitat became suitable for occupation (Soltis et al., 2006). Ecological niche modeling of other species of birds has also identified the Gulf Coast as a likely region of range disjunction at the LGM (e.g., Shipley et al., 2013).

### 3.4 Hybrid zone

**Figure 4:**
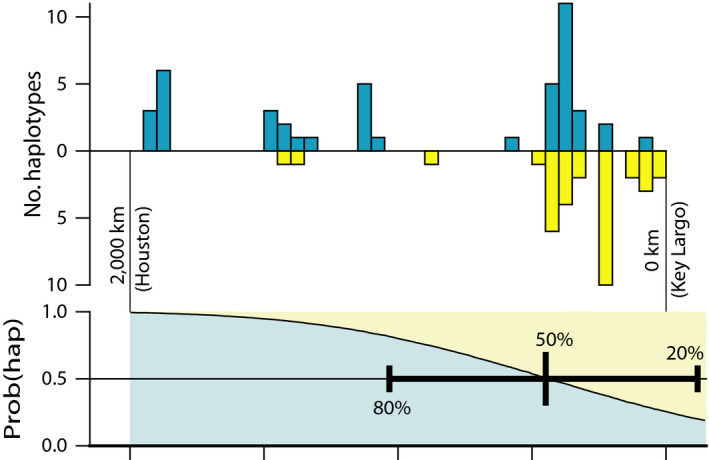
Upper: observed geographic distribution of eastern North American (blue) mtDNA haplotypes at the ND2 locus and those found predominantly in the Florida peninsula (yellow; e.g., Figure 1) along a transect from Key Largo (FL) to Houston (TX) in *B. lineatus*. Lower: inferred probability of observing eastern haplotypes along the same transect based on a logistic fit to a cumulative normal distribution. 20%, 50%, and 80% points in the distribution are indicated by vertical bars

A clade of three ND2 haplotypes was frequent in the Florida peninsula, but became increasingly uncommon to the north and west (Figure 1). Our ecological niche modeling suggests the habitat of *B. lineatus* was discontinuous near this area during the last glaciation. Because this pattern of genetic divergence in the Southeastern United States. Is generally consistent in many organisms and has been ascribed to Pleistocene allopatry followed by range expansion, parapatry, and introgression (e.g., Soltis et al., 2006), we modeled the current transition as a hybrid zone. The 78 haplotypes of individuals sampled in Florida, Louisiana, and Texas were each assigned “southeastern” or “common” designations and plotted against distance along a transect from Key Largo FL to Houston TX (Figure 4). A probit fit to a cumulative normal distribution, using an iterative maximum likelihood procedure, successfully converged and resulted in estimates of slope and intercept of 0.00145 and −0.6512, respectively. The center of the zone was estimated as 449 km NNW of Key Largo, near Ocala, FL; the maximum slope of the transition between the haplotype clades was estimated as 5.78 × 10^−4^ km^‐1^ and the 20%‐80% width of the zone as 1,158.6 km (Table 3, Figure 4).

**Table 3:** Characteristics of four avian hybrid zones in the Southeastern United States

Species	Plumage divergence	ND2 sequence divergence	Center of hybrid zone (km)^a^	Logistic width of hybrid zone (km)^b^
*Buteo lineatus*	Quantitative (color intensity)	0.0029	449 (S. Ocala)	1,158
*Strix varia*	None	0.0048	877 (Apalachicola)	844
*Melanerpes carolinus*	Discrete (forehead band)	0.0010	503 (N. Ocala)	435
*Poecile carolinensis*	None (e.g., Gill et al., 1999)	0.0423	1,248 (Tombigbee)	176

a Distance along transect from Key Largo, FL.

b 20%‐80% width.

### 3.5 Species limits

We used sequences of the mitochondrial ND2 gene, along with those of the two nuclear introns, to investigate consistency of gene trees inferred from the three loci with species status of eastern and western red‐shouldered hawks, based on a Bayesian coalescent analysis. The BPP analyses, using three differing sets of initial estimates of divergence time and effective population size, each replicated twice with differing initial seeds, and with adaptive adjustment of the MCMC step length, all resulted in posterior probabilities of 1.0 for separate species status of western and eastern populations. No attempt was made to assay the status of the Florida populations because the BPP program does not accommodate hybridization or continuing gene flow (Yang, 2015).

### 3.6 Interspecific comparisons

Barred owls (*Strix varia*), red‐bellied woodpeckers (*Melanerpes carolinus*), and Carolina chickadees (*Poecile carolinensis*) have geographic distributions in the Southeastern United States similar to that of *B. lineatus*. In addition, they all show patterns of southeast‐northwest genetic divergence that might be signatures of historical events common to all four species. Ecological niche modeling was not reported in those studies; however, all three are common residents of eastern deciduous forest where they are sympatric with the hawk. It seems probable that they possessed similar LGM geographical distributions.

Unfortunately, varying mitochondrial loci had been used in the original studies of these species. In order to have a common scale of differentiation, we sequenced exemplars of eastern and western individuals of *S. varia* and *P. carolinensis* for the ND2 gene to compare with our new results for *B. lineatus*, and with the previously published results for *M. carolinus*. In both cases, the tissues we sequenced were drawn from individuals used in the original studies. The new sequences have been deposited in GenBank (MK495851‐MK495854); the percent sequence divergence for 1,041 bp of ND2 for all four pairs of taxa is shown in Table 3. The divergence in *P. carolinensis* was an order of magnitude greater than that in the other three species.

**Figure 5:**
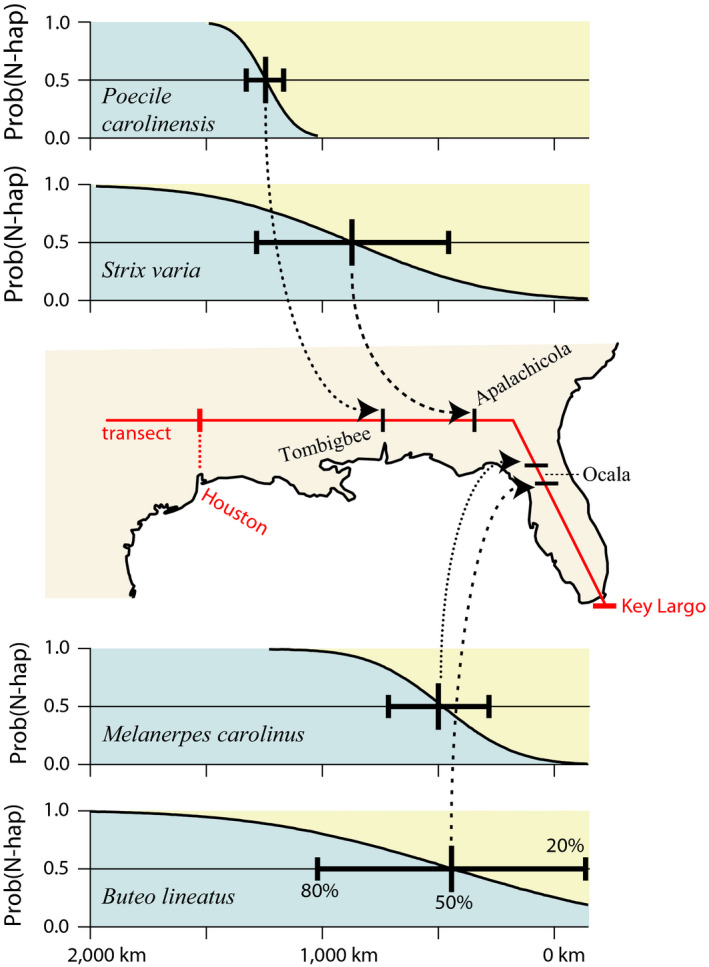
Logistic estimates of position and shape of four avian mitochondrial hybrid zones in the Southeastern United States. Central panel shows position of geographic transect from Key Largo (FL) to Houston (TX). Upper and lower panels show cumulative normal probability fits to empirical distributions of north or western (N‐hap) versus Florida peninsula or eastern haplotypes; center and 20%‐80% positions of distributions are indicated

In order to compare the geographical patterns of differentiation among the four species, we analyzed the zones of divergence in *S. varia* and *P. carolinensis* using the same approach we described above for *B. lineatus*, and which had previously been employed in a study of *M. carolinus*. In the case of *S. varia*, we used the mitochondrial control region haplotype frequencies reported for Florida, Louisiana, and Texas (Barrowclough et al., 2011) and projected them onto the same transect used for *B. lineatus*. The probit fit to a cumulative normal indicated a narrower, steeper hybrid zone than that for *B. lineatus*, with an approximate center near the Apalachicola River in the Florida panhandle (Table 3). For *P. carolinensis*, we used reported frequencies of mitochondrial restriction sites from Georgia, Alabama, Mississippi, Louisiana, and Texas (Gill et al., 1999, 2005), projected onto the *B. lineatus* transect, with a similar probit analysis. The results indicated a very narrow, steep transition with a center just west of the Tombigbee River in Mobile Co., Alabama (Table 3). For *M. carolinus*, the hybrid zone was narrower than that of *B. lineatus*, but with a center just 54 km north of that of the latter. A comparison of the geography of the zones for the four species is shown in Figure 5.

The estimated widths of the four hybrid zones differed by a factor of approximately 6.6. For neutral introgression, zone width is expected to scale with the magnitude of gene flow and the square root of time since initial contact (Crank, 1975; Endler, 1977). If the four species were all responding to habitat change associated only with the end of the last glacial cycle, then time would be approximately the same for the species. However, the species differ dramatically in their size, life history, and ecology; consequently, they might be expected to differ in the extent of their natal dispersal (gene flow). We found two studies of natal dispersal in *B. lineatus* that reported the standard error of mean dispersal distance and sample size for a total of three populations (Bloom, Scott, Papp, Thomas, & Kidd, 2011; Dykstra et al., 2004); these yielded estimates of root‐mean‐square natal dispersal distances of 22.9, 112.4, and 185.6 km; we used the median in further analyses. For *S. varia*, Livezey (2009) analyzed some band recovery data consistent with a root‐mean‐square total dispersal distance of 69 km. Cox and Kessler (2012) reported radio‐tracking natal dispersal data for *M. carolinus* equivalent to a gene flow distance of approximately 2 km. We were unable to locate any detailed dispersal studies providing the requisite dispersal data for *P. carolinensis*; the birds of North America account provides anecdotal distances of 1, 4, and 8 km (Mostrum, Curry, & Lohr, 2002); these are roughly consistent with estimates from populations of the closely related *Parus major* (e.g., Barrowclough, 1980); we used the median in a correlation analysis.

**Figure 6:**
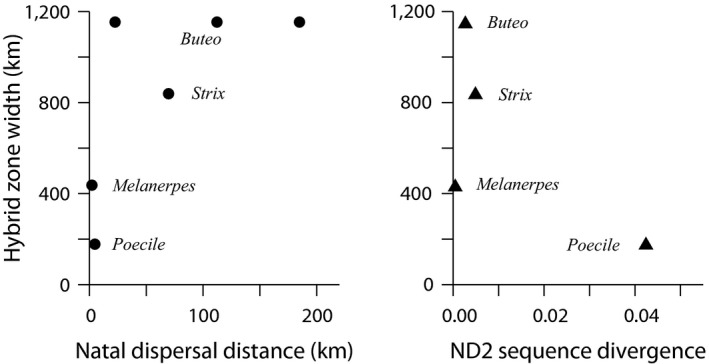
Scatter of estimates of 20%‐80% Florida/Gulf Coast hybrid zone widths for four species pairs of birds versus estimates of natal dispersal distances (left) and ND2 divergences (right)

We scattered our four estimates of hybrid zone width against both the estimates of dispersal distances and the estimates of ND2 divergence (Figure 6). Hybrid zone width scaled positively with the estimates of dispersal distance; the Pearson product moment correlation coefficient was 0.97. The hybrid zone width scaled weakly negative with ND2 divergence; the correlation coefficient was −0.70.

## 4 DISCUSSION

### 4.1 Eastern North America‐Pacific Coast divergence

We found major genetic divergence between the California samples of red‐shouldered hawks and those from the eastern portion of the range, consistent with species‐level taxa in a Bayesian coalescent analysis. At the ND2 locus, there was no sharing of haplotypes (Figure 1) and our estimate of *F*_st_, 0.65, indicates that two‐thirds of the genetic variance was distributed between the regions. For the two introns, the divergence was less, but there were salient differences in allelic frequencies between the regions and this was statistically significant for one of them (Table 2; Figure 2), consistent with differences expected between mitochondrial and nuclear divergence based on differences in their respective coalescent times (e.g., Zink & Barrowclough, 2008). For ND2, there was approximately an order of magnitude less nucleotide diversity in the California populations than in the eastern ones; there was also more variation in the east for both G3PDH and TGFB2. Those results are consistent with the major difference in range sizes of the two populations, presumably reflective of substantial differences in *N_e_* and, as a consequence, genetic variation. The patterns are also consistent with no recent gene flow between the two portions of the range. At present, they are completely allopatric and the ENM results suggest they may have been so during recent glacial cycles (Figure 3).

The genetic divergence between these eastern and western North America populations is not surprising; a previous microsatellite investigation had uncovered similar results (Hull et al., 2008) and the birds show phenotypic differences in plumage (Wheeler, 2018b).

### 4.2 Genetic variation in eastern populations

The salient feature found among the eastern population samples was the presence of a clade of ND2 haplotypes at high frequency in the Florida peninsula, decreasing in frequency with distance to the north and west. Little genetic variation could be attributed to populations within regions (Table 2). Given the differences in plumage between the peninsula birds and those to the north, as well as our historical niche modeling and the well‐known pattern of biotic differentiation in this area, we interpret these ND2 results as the signature of a zone of secondary contact between populations isolated from one another until after the LGM.

### 4.3 A hybrid zone in peninsula Florida

The discovery of genetic evidence for a zone of secondary contact in central Florida was surprising given prior taxonomic treatments of this hawk. For example, Mayr and Short (1970), in their systematic analysis of the North American avifauna, treated the southern Florida population as an uncomplicated, weakly to moderately differentiated subspecies, not posing any taxonomic concern at the species level. In part, the failure to recognize an important biogeographic signal resulted from a lack of quantification of the pattern of variation in plumage and size with geography. James (1970) suggested that wing length in this species (the Florida subspecies was originally based on size) was broadly clinal, but an examination of her Table 5 (i.e., p. 371) suggests it is actually quite steep, and possibly a step. Of course, with a few notable exceptions (e.g., Johnson, 1980; Rising, 2001), most species of birds have never been subjected to modern statistical analyses of geographical variation in size, shape, or plumage color (Zink & Remsen, 1986).

An alternative hypothesis to our suggestion of secondary contact in these hawks might be that this hybrid zone represents a case of primary divergence. However, several qualitative arguments suggest that is not the most parsimonious scenario. First, if the divergence did begin post‐LGM, then there has been less than 20,000 years to achieve a near fixation of a newly arisen haplotype in a large population. More saliently, plumage differences in the hawk populations are coincident with unlinked mitochondrial differences, as well as with the position of LGM refugia and the geographical patterns of multiple additional species (see below).

Our estimate of the width of the hybrid zone is only approximate. We did obtain statistical convergence of estimates of the width and shape of the hybrid zone (Figure 4). However, there were few samples to the immediate north of the estimated center of the zone; consequently, the width may be somewhat narrower than our estimate. Additional samples of these hawks from northern Florida and the Southeastern United States would improve our confidence in its width.

### 4.4 Classification of the *Buteo lineatus* complex

The California population of this hawk was originally described as a distinct species, *B. elegans* (Cassin, 1855). Our results indicate it is genetically divergent from the eastern ones on the basis of both mitochondrial and nuclear DNA (Table 2). A previous survey of 21 microsatellite loci had demonstrated 100% discrimination between California and eastern populations (Hull et al., 2008). The overall evidence suggests that not only is there no current gene flow between these regions but that there has not been any for a sufficient period of time to allow the California population to become monophyletic (e.g., Figure 3). It is expected to take approximately 2*N_e_* generations for neutral coalescence of mitochondrial genes to occur; the results of our coalescent analysis using the BPP program were consistent with that interpretation. The niche modeling indicates the period of isolation must have been well in excess of 20,000 years, that is, it must pre‐date the last glacial cycle, that is, 100,000 years. The ND2 sequences render the eastern and western populations 100% diagnosable. Morphologically, the Pacific Coast birds are also distinguishable on the basis of a much richer rufous coloration, particularly on the undersides; they also may be nearly 100% diagnosable on the basis of the number and size of tail bands (Crocoll, 1994; Johnson & Peeters, 1963; Palmer, 1988; Pyle, 2008). *B. eleg*ans clearly represents a phylogenetic species and is an appropriate taxon for phylogenetic and biogeographic investigation.

Red‐shouldered hawks found in the central and southern portion of the Florida peninsula, described as *B. l. extimus* by Bangs (1920), are substantially smaller than those to the north (e.g., James, 1970) and much paler in color (Wheeler, 2018a). Eighteen percent of the mtDNA variation was distributed between the peninsula and the rest of the eastern range of the bird. At present, there appears to be extensive introgression in the peninsula region, with a broad hybrid zone, but the ecological niche modeling suggests this is a postglacial phenomenon in a species with substantial dispersal ability. Advocates of the biological species concept will regard this taxon as a well‐differentiated subspecies (e.g., Dickinson & Remsen, 2013). However, our opinion is that the Florida peninsula population has had a separate evolutionary history from that of the other eastern birds and consequently represents an appropriate unit for studies of diversification and historical biogeography; therefore, it represents a phylogenetic species. This would not be apparent were the taxon to be simply regarded as a subspecies in the *B. lineatus* complex. For example, Dickinson and Remsen (2013) recognize additional subspecies in that complex that cannot be historical units given that their current, largely latitudinal, ranges (Figure 1) are at odds with the glacial history of eastern North America. Ranking *B. extimus* as a subspecies of *B. lineatus* would result in treating historical entities and ahistorical classes as equivalents, thus resulting in a nonhierarchical classification, useless for historical investigation.

### 4.5 Comparative avian phylogeography in the Southeastern United States

Four avian taxa have now been identified showing mtDNA divergence with hybridization on the Florida peninsula or along the Gulf Coast of the southern United States. This is a region identified as a suture zone, on the basis of external morphology of many organisms (Remington, 1968). Three of those four hybrid zones, all except that of the Carolina chickadee, are centered within the geographical limits of Remington's original description of the zone. A survey of molecular studies (Soltis et al., 2006) showed that a wide variety of organisms are genetically divergent in the same region. The four avian zones differ in degree of morphological and mtDNA divergence, position of the center, and width of the zone (Table 3, Figure 5). However, these may not necessarily represent responses to differing ultimate causes; for example, three Pleistocene refugia in southern Europe have led to multiple patterns of range expansion and subsequent hybridization (Hewitt, 1999).

Of interest is whether these four avian zones represent patterns resulting from a single temporal biogeographic event. This would appear to be unlikely; the extent of mtDNA divergence, often thought to be a surrogate for time, differed by more than an order of magnitude among the species pairs. That suggests that some of the taxa, particularly *P. carolinensis*, may have differentiated through repeated isolation events over multiple glacial cycles; Soltis et al. (2006) indicated that scenario might be a general phenomenon.

Secondly, the centers of the zones differ substantially in geographical position. The *Poecile* and *Strix* cases would appear to correspond to the Tombigbee and Apalachicola discontinuities of Soltis et al. (2006). Barton (1979) and Barton and Hewitt (1985) pointed out that hybrid zones are attracted to physical barriers and density troughs such as those presented by wide rivers. The *Melanerpes* and *Buteo* zones centered on the Florida peninsula seem to be exemplars of the southern edge of the canonical Northern Florida Suture Zone. Remington (1968) suggested the zone represented the position of initial contact for many previously allopatric species pairs. In an important synthesis of phylogeographic patterns found in turtles of this same region, Walker and Avise (1998) discovered exemplars of each of the geographic patterns we observed in the four birds.

Finally, the widths of the four hybrid zones vary by a factor in excess of six. The widths of neutral hybrid zones are expected to scale with dispersal (gene flow) distance and the square root of time since secondary contact (Endler, 1977). The latter is likely similar for these species. However, dispersal distances vary greatly among the species pairs, and the hybrid zone widths scatter reasonably well with those estimates (Figure 6). For hybrid zones maintained by a balance between dispersal and selection, the widths should scale with the inverse square root of selection intensity (Barton & Hewitt, 1985). In these four cases, widths scale poorly with phenotypic differences (e.g., sexual selection) and only weakly with genetic divergence (Figure 6, Table 3), possible correlates of general selective divergence. Thus, it appears that these avian zones have been in isolation for varying numbers of glacial cycles and that their current positions and widths reflect their idiosyncratic dispersal propensities. Further insight will require denser geographical sampling, extensive nuclear sequencing, and examination of additional species pairs.

Overall, these four avian cases, along with numerous examples from plants and other animals (Soltis et al., 2006), suggest there may be additional, cryptic divergence waiting to be discovered in the avifauna of the Southeastern United States. Interestingly, Remington (1968) and later Newton (2003), suggested six species of birds as candidates for differentiation in the Northern Florida Suture Zone. Three of those (northern bobwhite, wild turkey, and Bachman's sparrow) have now been surveyed using phylogeographic methods, and none were found to possess significant mitochondrial differentiation. Thus, it appears that predicting which avian taxa might be involved is not transparent. Nevertheless, species that are permanent residents or only partially migratory would seem to be particularly appropriate candidates for further investigation.

## CONFLICT OF INTEREST

The authors have no conflict of interest.

## AUTHOR CONTRIBUTIONS

G.F.B. and J.G.G. designed this study; W.M.M. collected the data; G.F.B., J.G.G., and M.E.B. performed the analyses; and G.F.B. wrote the paper.

## Data Availability

All DNA sequences and their associated specimen localities and museum provenances are provided in the GenBank accessions (MK495851‐MK495854; MK523769‐MK524014; MK551872‐MK552100; MK575607‐MK575736). Arlequin, BPP, MAXENT, and SAS datafiles and scripts are available at https://doi.org/10.5061/dryad.1q33s69.
